# Optimized Rear‐Interface Passivation of SnS Thin‐Film Solar Cells Using a Controlled Germanium Oxide Interlayer for Enhanced Photovoltaic Performance

**DOI:** 10.1002/smll.202507626

**Published:** 2025-09-19

**Authors:** Rahul K. Yadav, Vishesh Manjunath, Yong Tae Kim, Girish U. Kamble, Wookyung Jeon, Parag R. Patil, Neha Bisht, Jin Hyeok Kim, Yohan Yoon, Jaeyeong Heo

**Affiliations:** ^1^ Department of Materials Science and Engineering and Optoelectronics Convergence Research Center Chonnam National University Gwangju 61186 Republic of Korea; ^2^ Department of Materials Engineering Korea Aerospace University Goyang 10540 Republic of Korea

**Keywords:** DLTS, germanium oxide (GeO_x_), MoS_2_ layer formation, Na diffusion, rear interface passivation, tin sulfide (SnS)

## Abstract

Tin monosulfide (SnS) holds significant promise as a sustainable, earth‐abundant absorber for thin‐film solar cells (TFSCs), however, device efficiencies remain hindered by detrimental interfacial quality at the rear contact. Defect states, interfacial reactions, and uncontrolled alkali diffusion at the Mo/SnS interface introduce severe recombination losses, limiting photovoltaic (PV) performance. Here, a tailored back‐interface engineering strategy employing a thermally evaporated and controlled oxidized Germanium (germanium oxide GeO_x_) interlayer to passivate the Mo/SnS interface is reported. This compact, chemically stable GeO_x_ interlayer simultaneously improves absorber morphology, passivates deep‐level defects, suppresses sodium (Na^+^) diffusion from the substrate, and inhibits MoS_2_ formation during thermal processing. Deep‐level transient spectroscopy (DLTS) analysis confirms a significant reduction in mid‐gap donor‐like traps associated with Na‐induced defects, thereby mitigating non‐radiative recombination pathways. These synergistic effects collectively lead to a substantial enhancement in power conversion efficiency, increasing from 3.71% in the control device to 4.81% in the GeO_x_‐modified device having device stack of SLG/Mo (800 nm)/GeO_x_ (7 nm)/SnS (1500 nm)/CdS (50 nm)/i‐ZnO (50 nm)/AZO (400 nm)/Al (1000 nm). Overall, this study highlights the potential of GeO_x_ as an effective interfacial modifier for SnS PVs, offering a practical strategy to overcome longstanding limitations in device performance.

## Graphical abstract

Tin monosulfide (SnS) thin‐film solar cells suffer from back contact losses, limiting efficiency. Introducing a scalable GeO_x_ interlayer between Mo and SnS improves absorber morphology, suppresses defects, and blocks detrimental diffusion. This rear contact passivation enhances device performance, achieving 4.81% efficiency, advancing sustainable photovoltaics with earth‐abundant, non‐toxic materials.

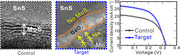

## 1 Introduction

Over the past few decades, advancements in thin‐film solar cells (TFSCs) have notably improved the viability and competitiveness of photovoltaic (PV) technologies for large‐scale energy production.^[^ ^1^ ^]^ Among the PV systems developed to date, conventional crystalline Si (c‐Si) and second‐generation thin‐film technologies such as Cu(In,Ga)Se_2_ (CIGS) and CdTe have made substantial strides, nearing their theoretical power conversion efficiency (PCE) limits.^[^ ^2^, ^3^, ^4^, ^5^ ^]^ Notably, the theoretical PCE of a single‐junction solar cell is limited to a maximum of ≈33% under terrestrial conditions, a value known as the Shockley‐Queisser limit.^[^ ^6^ ^]^ However, the high manufacturing costs of c‐Si, combined with the scarcity and toxicity of elements such as In, Ga, and Cd, present notable challenges for the large‐scale deployment of the aforementioned technologies.^[^ ^7^, ^8^ ^]^ Consequently, exploring alternative low‐cost, earth‐abundant, and non‐toxic absorbers is crucial for enabling the production of larger‐area panels and achieving grid parity.

Among emerging PV absorbers, tin sulfide (SnS) has attracted significant interest due to its earth‐abundant, non‐toxic elements and favorable optoelectronic properties. SnS can crystallize in orthorhombic, cubic, or hexagonal phases, with the orthorhombic phase being thermodynamically stable and most suitable for PVs owing to its optimal bandgap (E_g_) (≈1.3 eV) and anisotropic carrier transport. Although SnS possesses a simple binary composition and exhibits promising intrinsic properties, orthorhombic SnS solar cells encounter several persistent limitations. For instance, the naturally occurring layered crystal structure of SnS promotes anisotropic growth, typically leading to plate‐like morphologies prone to void formation and substantial voltage loss.^[^ ^9^ ^]^ In our previous reports, we have deposited SnS absorbers using VTD at a high deposition rate of 75 nm min^‒1^, a condition shown to favor cube‐like morphology. Such specific condition suppresses the formation of inherent plate‐like morphology of orthorhombic SnS, thereby ensuring structural stability and electronic quality for subsequent interface engineering and device optimization.^[^ ^9^, ^10^, ^11^ ^]^ Furthermore, the best‐performing SnS‐based devices currently achieve PCEs of ≈4.0%–4.5%, with few reports exceeding this threshold.^[^ ^12^, ^13^, ^14^, ^15^ ^]^ These inferior efficiencies arise from a combination of factors, including poor band alignment, high interfacial defect densities, reduced minority carrier lifetimes, and substantial non‐radiative recombination losses.^[^ ^16^, ^17^, ^18^, ^19^, ^20^, ^21^ ^]^ In particular, the back contact interface has emerged as a critical bottleneck, where severe non‐radiative recombination notably impairs charge extraction.^[^ ^22^, ^23^, ^24^, ^25^ ^]^ Recent studies have highlighted that optimizing the back interface by tailoring energy band alignment, minimizing interfacial defects, and introducing passivating interlayers can substantially suppress recombination losses and enhance device performance.^[^ ^26^, ^27^, ^28^, ^29^ ^]^

A major contributor to the PCE losses observed in chalcogenide TFSCs is the presence of suboptimal interfaces, particularly at the rear interface (typically molybdenum (Mo)) and at the heterojunction between the absorber and buffer layers.^[^ ^30^, ^31^, ^32^, ^33^ ^]^ Back‐contact recombination is typically more pronounced due to the high defect density (dangling bonds, vacancies) at the absorber–metal interface, where photogenerated minority carriers having traversed the full absorber thickness are readily captured. While front interfaces are often passivated with selective buffer/window layers, rear contacts generally lack such optimization, making them dominant recombination sites in the absence of targeted passivation or band‐engineering, thereby limiting open‐circuit voltage (*V*_OC_) and overall efficiency.^[^ ^34^ ^]^ These poor‐quality interfaces promote substantial non‐radiative recombination, particularly at the rear interface, which considerably reduces device efficiency.^[^ ^35^, ^36^, ^37^, ^38^ ^]^ To address this rear interface recombination in well‐developed c‐Si‐, CIGS‐, and CdTe‐based solar cells, an effective strategy involves incorporating a dielectric passivation layer.^[^ ^39^, ^40^, ^41^, ^42^ ^]^ Several metal oxides, including SiO_2_, Al_2_O_3_, TiO_2_, and HfO_2_, have been employed as rear‐interface passivation layers in CIGS and CdTe solar cells, yielding significant gains in carrier lifetime and device efficiency. For example, an 8 nm SiO_2_ layer increased CIGS PCE from 11.9% to 13.2%, a 25 nm Al_2_O_3_ layer from 8.1% to 9.8%, and a 40 nm HfO_2_ layer from 10% to 11.8%, while a 6 nm amorphous TiO_2_ layer boosted CIGS efficiency from 1.65% to 8.76%. In CdTe, a 1 nm ALD‐deposited Al_2_O_3_ layer improved PCE from 10.7% to 12.1%. These results underscore the critical influence of oxide thickness and composition in optimizing interface passivation and enhancing overall device performance.^[^ ^43^, ^44^, ^45^, ^46^, ^47^ ^]^ A similar back‐contact modification strategy has also been applied to antimony selenide (Sb_2_Se_3_) solar cells, where insertion of ultrathin passivation layers at the rear interface has been shown to suppress non‐radiative recombination and enhance device performance. Insertion of a 15 nm WO_3_ layer improved PCE from 7.41% to 9.24%, while a 5 nm PbSe layer enhanced PCE from 3.04% to 8.43%. These findings demonstrate the strong influence of interfacial engineering on mitigating recombination losses and boosting Sb_2_Se_3_ device efficiency.^[^ ^26^, ^48^ ^]^ Moreover, interfacial engineering techniques have been successfully implemented in perovskite solar cells to reduce interfacial recombination and modulate film crystallization, demonstrating the broad applicability and relevance of this approach across emerging PV technologies.^[^ ^49^, ^50^, ^51^ ^]^

Despite the demonstrated success of rear interface engineering in other thin‐film technologies, the application of rear surface passivation and recombination suppression in SnS‐based TFSCs remains largely unexplored. A key challenge in this application arises from the diffusion of excess Na⁺ ions from soda‐lime glass (SLG) substrates and from decomposition reactions occurring at high temperatures during vapor‐transport deposition (VTD) growth, which promote the formation of undesirable interfacial layers such as MoS_2_ and Na_2_S at the Mo/SnS interface.^[^ ^12^ ^]^ Consequently, charge transfer at the back contact is limited by these redundant interfacial layers, leading to increased non‐radiative recombination and impaired device performance.

Sub‐stoichiometric germanium oxide (GeO_x_) has gained significant attention for its ability to form stable, self‐passivating interlayers that effectively suppress interface states in semiconductor devices. Owing to its tunable stoichiometry, suitable band alignment, and good thermal stability, GeO_x_ serves as an efficient passivation layer for enhancing interfacial quality and reducing defect‐mediated recombination. These properties make GeO_x_ highly attractive for applications in thin‐film PVs. Given this background, a novel strategy to address improper contact and recombination losses at the back interface of planar heterojunction SnS solar cells was developed herein. Specifically, a thin film of Ge was deposited over SLG/Mo substrates, with the Ge layer intended to passivate the rear interface.^[^ ^52^, ^53^ ^]^ Considering the thickness‐dependent properties of the passivation layer, an ultra‐thin Ge layer was initially applied. Upon air exposure, elemental Ge readily and randomly forms a sub‐stoichiometric native oxide (GeO_x_, 1 < × < 2), which is structurally and chemically less stable than stoichiometric GeO_2_. Nevertheless, several studies have demonstrated that a controlled thin GeO_x_ interlayers could provide beneficial passivation at semiconductor interfaces, typically reducing interface state densities and lowering surface recombination velocities.^[^ ^54^, ^55^ ^]^ Thus, to exploit this advantage, we systematically controlled the thickness of the Ge layer and its subsequent oxidation to develop a stable and uniform GeO_x_ interfacial layer. In addition to rear interface passivation, the GeO_x_ interlayer facilitated the growth of larger SnS grains, inhibited the diffusion of Na⁺ ions, and suppressed the formation of an undesired MoS_2_ layer at the rear interface. A maximum PCE of 4.81% was achieved in target devices configured as SLG/Mo/GeO_x_/SnS/CdS/i‐ZnO/AZO/Al, representing a substantial advancement for SnS‐based thin‐film PVs. Overall, this study represents the first demonstration of effective rear interface passivation in SnS solar cells and provides a new pathway for addressing interfacial recombination and absorber crystallinity through targeted back‐interface modification.

## 2 Results and Discussion

**Figure 1:**
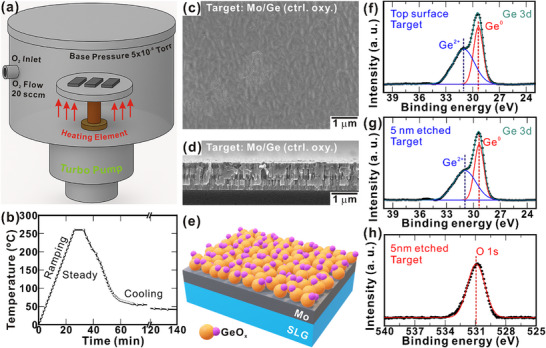
a) Schematic of the oxidation chamber. b) Temperature profile over time during controlled oxidation. c) Top surface, d) cross‐sectional morphology, and e) schematic of SLG/Mo/Ge after controlled oxidation. f) XPS 3d peak of Ge at the surface. g) XPS 3d peak of Ge at 5 nm depth. h) XPS 1s peak of O at 5 nm depth.

To elucidate the influence of rear interface passivation on the performance of VTD‐grown SnS TFSCs, we systematically examined a modified back‐contact structure comprising SLG/Mo/Ge and compared it with the conventional SLG/Mo configuration. To fabricate the SLG/Mo/Ge sample, a thin Ge layer was thermally evaporated onto a cleaned SLG/Mo substrate, serving as the interfacial layer. Subsequently, the Ge interfacial layer was thermally oxidized in a controlled environment, as illustrated in **Figure** **1**a.

Before the controlled oxidation process, determining the appropriate Ge film thickness on Mo was considered critical for optimizing device performance. To identify this optimal thickness, devices were fabricated using Mo substrates coated with Ge layers of varying thicknesses (1–10 nm) without being subjected to controlled oxidation (Figure S1, Supporting Information). PV parameters, including *V*_OC_ and short‐circuit current density (*J*_SC_), improved with increasing Ge thickness up to 7 nm, while the fill factor (FF) continuously declined. When the Ge thickness exceeded 7 nm, both *V*_OC_ and *J*_SC_ decreased, leading to deteriorated device performance. Figure S2 (Supporting Information) presents the light current density‐voltage (*J*–*V*) curves of devices fabricated with different Ge thicknesses. Among these devices, the SLG/Mo/7‐nm‐thick Ge layer configuration exhibited the best performance, achieving a PCE of 2.56%, *V*_OC_ of 0.289 V, *J*_SC_ of 26.95 mA cm^−2^, and FF of 32.83%. Nonetheless, this performance, although superior among the Ge‐containing devices, remained considerably poorer than that of the reference SLG/Mo device without Ge (Figure S3, Supporting Information).

To further investigate the deterioration in device performance associated with the Ge interfacial layer, we conducted morphological, structural, and compositional analyses. Figure S4a,b (Supporting Information) present the surface and cross‐sectional morphologies of the SLG/Mo substrate and its counterpart coated with a 7‐nm‐thick Ge thin film via thermal evaporation. Notably, the SLG/Mo/Ge sample exhibits non‐uniform surface coverage of Ge film (Figure S4b, Supporting Information). Grazing‐incidence X‐ray diffraction (GI‐XRD) patterns (Figure S5a,b, Supporting Information) reveal no discernible peaks corresponding to crystalline Ge, confirming its amorphous nature. Additionally, the characteristic peaks of Mo (110), (200), and (211) appear attenuated following Ge deposition. X‐ray photoelectron spectroscopy (XPS) results for the top surface of the 7‐nm‐thick Ge film thermally deposited onto Mo and at a depth of 5 nm are presented in Figure S6a,b (Supporting Information). Notably, the 3d XPS spectrum of Ge displays three distinct peaks at 32.9, 31.4, and 29.7 eV, corresponding to Ge^4+^, Ge^2+^, and Ge^0^, respectively. At a depth of 5 nm, Ge^2+^ and Ge^4+^ exhibit negative binding energy shifts of 0.4 and 0.1 eV, respectively, indicating reduced oxidation of Ge at greater depth. The single O 1s peak at 530.9 eV confirms the exclusive presence of Ge–O bonding. These results indicate substantial and uncontrolled oxidation of the Ge film upon air exposure, resulting in an uneven, mixed‐phase oxide layer composed of GeO, GeO_2_, and residual elemental Ge (GeO_x_). This finding aligns with previous reports describing rapid and non‐uniform oxidation of Ge thin films under ambient conditions.^[^ ^56^, ^57^, ^58^, ^59^ ^]^ The formation of such an uncontrolled oxide layer not only increases interfacial resistance but also disrupts charge transport across the rear interface, ultimately compromising device performance. Moreover, the total thickness of this oxidized intermediate layer likely exceeds the optimal limit, acting as a barrier rather than a passivation layer.^[^ ^60^, ^61^, ^62^ ^]^

To overcome the limitations associated with uncontrolled ambient oxidation of Ge interlayer, we aimed to engineer a stable and conformal oxide layer through a controlled oxidation process, as shown in Figure 1a. Oxidation of 7 nm‐thick Ge layer was conducted at temperatures ranging from 200 to 290 °C for 2 min, resulting in measurable performance gains (Figure S7, Supporting Information). As indicated in Table S1 (Supporting Information), all PV parameters, particularly *J*_SC_ and *FF*, steadily improved with increasing oxidation temperature, reaching a peak PCE of 4.24% at 260 °C. This finding suggests that elevated temperatures promote the formation of a more uniform GeO_x_ interfacial layer. Subsequently, the oxidation time was varied from 1 to 10 min while maintaining the optimal temperature of 260 °C. The corresponding device characteristics are presented in Figure S8 and Table S2 (Supporting Information). With increasing oxidation duration, both *J*_SC_ and *FF* continued to improve, whereas a slight decrease in *V*_OC_ was observed. This decline in *V*_OC_ is attributed to the increased thickness of the oxide layer resulting from oxide overgrowth. Oxide overgrowth refers to the formation of a surface oxide layer whose thickness exceeds the passivation optimum, typically due to oxidation durations or conditions beyond the kinetic regime that Favors defect suppression. This excessive oxide growth can alter band alignment, increase interfacial resistivity, and introduce additional defect states, thereby enhancing carrier recombination losses. Optimal performance with good cell‐to‐cell uniformity was achieved with an oxidation duration of 5 min, yielding a maximum PCE of 4.81%. Notably, this represents nearly a 30% improvement over the control device. The light *J‒V* characteristics of the best‐performing devices during controlled oxidation optimization at different temperatures and times are illustrated in Figure S9 (Supporting Information).

The temperature profile of the controlled oxidation process is illustrated in Figure 1b. The surface and cross‐sectional morphologies of the Ge film after controlled oxidation (7 nm; 260 °C; 5 min) are illustrated in Figure 1c,d, while Figure 1e presents a schematic of the SLG/Mo/GeO_x_ (controlled oxidation) substrate. The surface morphology of the Ge film becomes more compact and smoother following controlled oxidation. To probe the chemical state of the controlled GeO_x_ film, XPS analysis was performed at different etching depths. The Ge 3d core‐level spectra at the surface (1 nm etch) and at a 5 nm depth (Figure 1f,g) confirm the presence of Ge^2+^ and Ge^0^ species at 31.0 and 29.4 eV, respectively. Correspondingly, the O 1s spectra at 5 nm depth (Figure 1h) and at the surface (Figure S10, Supporting Information) display features characteristic of Ge–O bonding, validating the formation of a homogeneous GeO_x_ film with some residual elemental Ge. A thin GeO_x_ layer on the Ge surface, rather than a fully stoichiometric GeO_2_, effectively suppress carrier recombination at the back contact and enhance charge collection. XPS depth profiling of the 7 nm thermally evaporated Ge film shows a near‐stoichiometric Ge:O ratio (≈1:1) at the surface, gradually decreasing to ≈1:0.4 at a depth of 5 nm. Considering a linearly graded Ge sub‐oxide as discussed in Note S1a (Supporting Information), the calculated thickness of entire GeO_x_ region would be ≈9.5 nm. The expected increment in GeO_x_ layer after oxidation is ≈2.5 nm. Following the successful formation of this conformal and stable GeO_x_ interfacial layer, the modified SLG/Mo/GeO_x_ substrate was designated as the target device, while the Mo substrate (SLG/Mo) served as the control device. These designations were consistently used throughout the study to distinguish between devices fabricated on passivated and non‐passivated rear interfaces.

**Figure 2:**
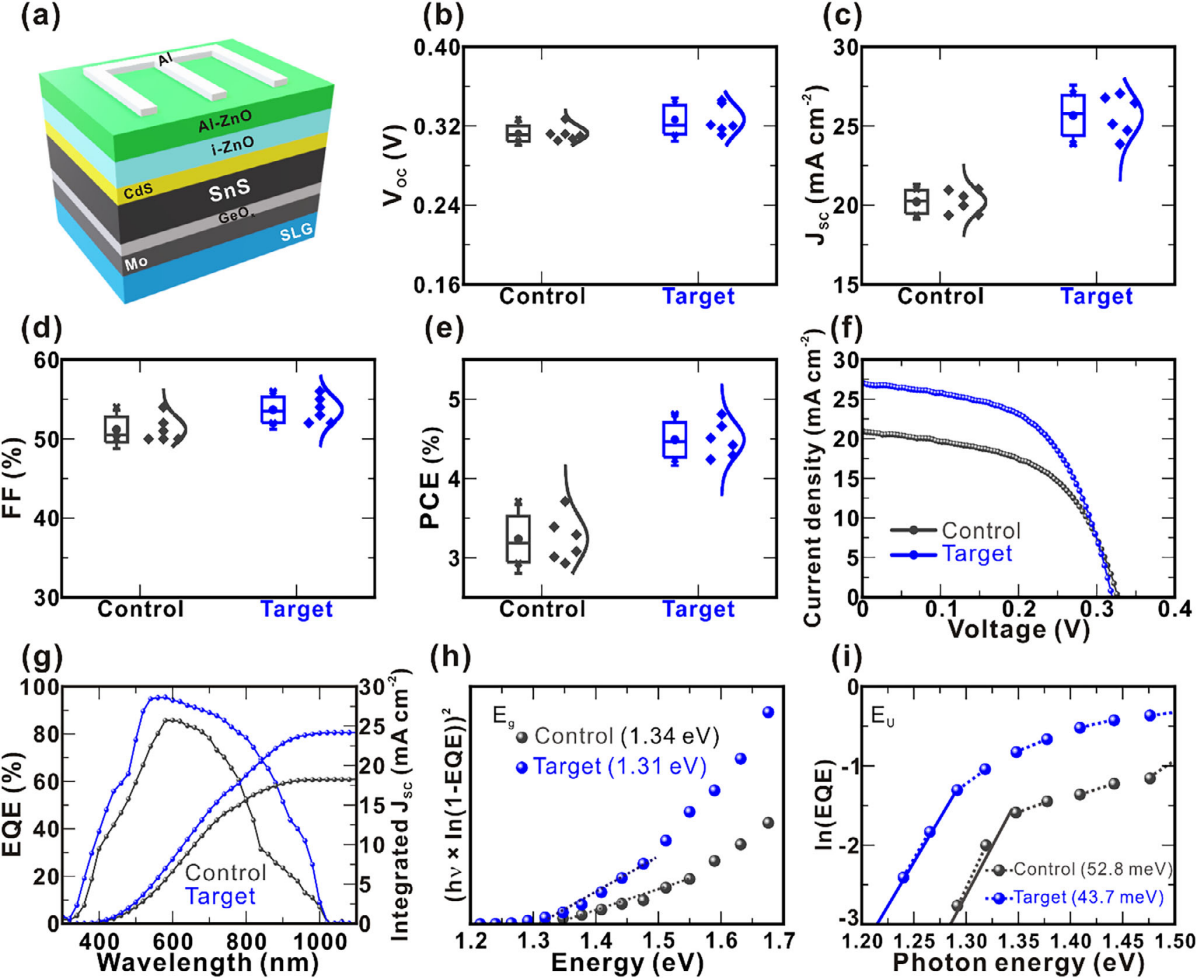
a) Schematic of the target device with the SLG/Mo/GeO_x_ configuration. Box plots of PV parameters for the control and target devices: b) *V*_OC_, c) *J*_SC_, d) *FF*, and e) PCE. Characterization of control and target devices: f) Light *J*–*V* characteristics, g) EQE spectra, and h) *E*_g_ and i) E_U_ extracted from the EQE spectra.

**Figure** **2**a presents a schematic of the fabricated target device, featuring a modified rear interface with a GeO_x_ interlayer formed by controlled oxidation. The statistical distributions of PV parameters for the control (SLG/Mo) and target (SLG/Mo/GeO_x_) devices are illustrated in Figure 2b–e. Notably, the target devices consistently outperform the control devices across all key parameters, highlighting the beneficial effect of rear GeO_x_ passivation. Figure 2f displays the *J‒V* characteristics of the best‐performing devices from both groups. The best‐performing control device achieved a PCE of 3.71%, with a *V*_OC_ of 0.327 V, a *J*_SC_ of 20.94 mA cm^‒2^, and an *FF* of 54.20%. In contrast, the best‐performing target device exhibited a notably improved PCE of 4.81%, with a *J*_SC_ of 27.00 mA cm^‒2^, an *FF* of 56.00%, and a slightly lower *V*_OC_ of 0.319 V. **Table** **1** summarizes the PV parameters of the optimized devices. Figure 2g presents the external quantum efficiency (EQE) spectra of the control and target devices, along with their respective integrated *J*_SC_ values. The integrated values of 24.27 and 18.25 mA cm^‒2^ for target and control, respectively, agree with those obtained from *J‒V* measurements, thus validating the trends observed in device performance. The observed discrepancy between the integrated *J*_SC_ values and those derived from *J‒V* measurements can be attributed to variations in the optical spectrum and the transmission characteristics of filters used during EQE measurements.^[^ ^61^, ^63^ ^]^ The enhanced EQE response of the target device across the full spectral range highlights the improved collection of photogenerated carriers enabled by the GeO_x_‐modified back interface. To further investigate the influence of back‐contact modification on the absorber layer, the *E*_g_ of the SnS films was estimated from Tauc plots derived from the EQE spectra (Figure 2h). Notably, the Urbach energy (*E*_U_), extracted from the EQE spectra (Figure 2i), was lower for the target device than for the control. This reduction in the *E*_U_ indicates decreased disorder and fewer sub‐bandgap states, suggesting suppressed non‐radiative recombination near the back interface, based on current comparison of control and target devices.^[^ ^26^, ^61^ ^]^ Moreover, the improved band‐tail characteristics further confirm the effectiveness of the GeO_x_ interlayer in passivating deep‐level defects. The following section presents an in‐depth analysis of the structural, morphological, and device properties of the control and target absorbers.

**Table 1:** PV parameters of best‐performing devices with (target) and without (control) modified back interfaces.

Device	Cell performance			
	*V*_OC_ [V]	*J*_SC_ [mA cm^‒2^]	*FF* [%]	PCE [%]
Control	0.327 (0.312 ± 0.007)	20.94 (20.22 ± 0.74)	54.2 (52.22 ± 1.60)	3.71 (3.23 ± 0.29)
Target	0.319 (0.326 ± 0.014)	27.00 (25.70 ± 1.27)	56.0 (54.72 ± 1.63)	4.81 (4.50 ± 0.22)

* The average and standard deviation values are presented in brackets.

**Figure 3:**
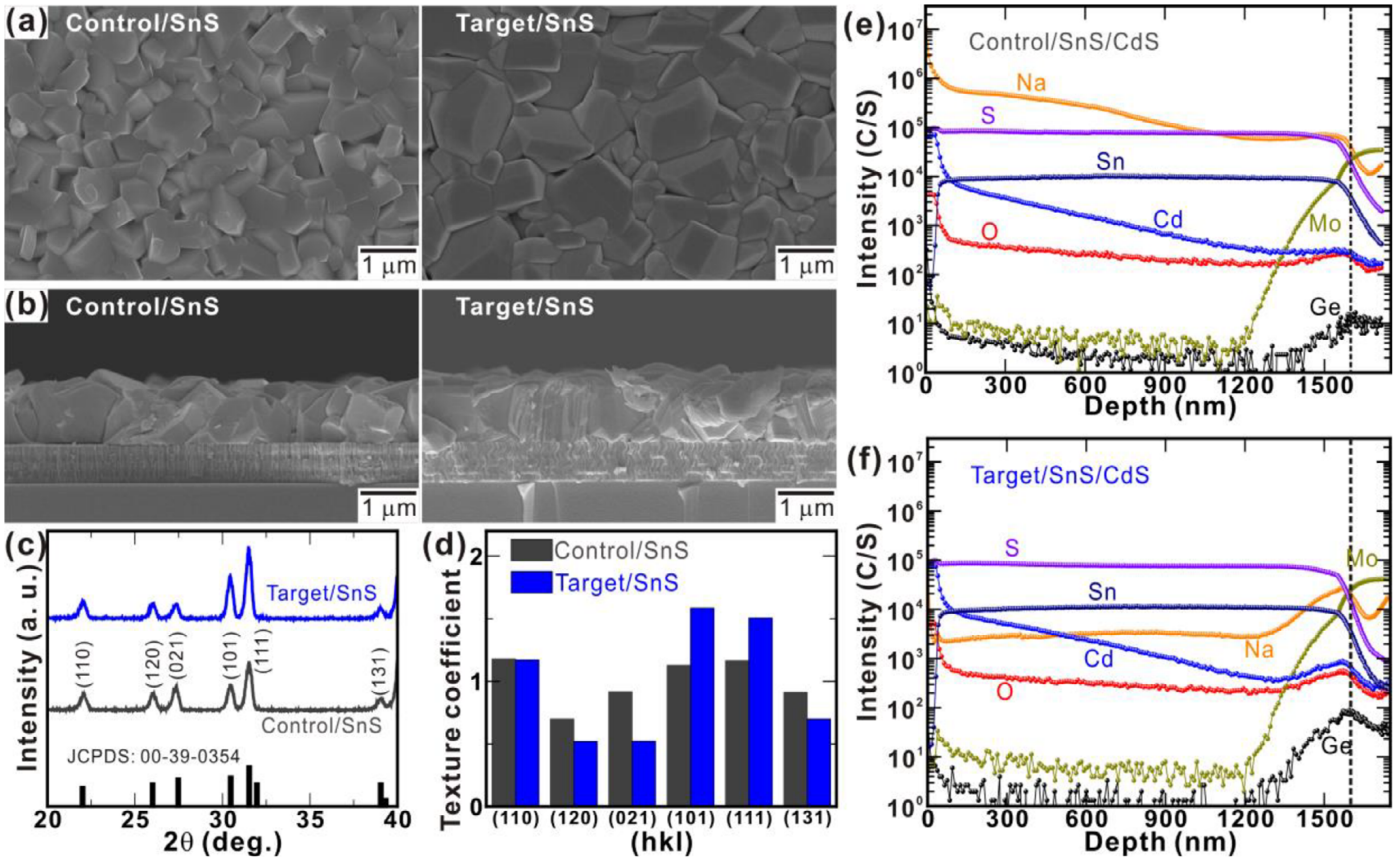
a) Top‐surface and b) cross‐sectional FE‐SEM images of the control and target SnS films. c) XRD patterns of the control and target films along with the JCPDS reference (00‐39‐0354). d) TCs for different crystal planes of the control and target films. e,f) SIMS depth profiles of the control and target films, respectively.

To better understand the performance differences between devices with and without back‐interface modification, we examined the crystal structures and surface morphologies of VTD‐grown SnS absorber layers deposited on both substrates. **Figure** **3**a,b respectively illustrate the field emission scanning electron microscopy (FE‐SEM) images of the top surface and cross‐section of SnS films grown on the two substrates. Notably, both absorber films display a compact morphology characterized by cube‐like grains (Figure 3a). However, the target film grown on the GeO_x_‐modified back interface presents a noticeably larger grain size than the control. Given that the deposition parameters (temperature, pressure, atmosphere, and duration) remain identical for both groups, the observed enhancement in grain size is primarily attributed to the presence of the GeO_x_ interfacial layer. Specifically, an optimized GeO_x_ layer enhances the grain growth and crystallinity of VTD‐grown SnS by either modifying the substrate surface properties or regulating the diffusion of Na⁺ ions from the SLG substrate into the SnS absorber. Numerous studies have demonstrated that Na^+^ diffusion from SLG substrates can critically influence grain growth in chalcogenide materials such as kesterites.^[^ ^60^, ^64^, ^65^ ^]^ As depicted in the cross‐sectional FE‐SEM image in Figure 3b, the control film exhibits a randomly oriented microstructure. In comparison, the target film displays denser and flatter morphology with a distinct preference for tilted grain growth, resulting in more compact and pinhole‐free morphology. The observed increase in grain size and the development of a preferred crystallographic orientation in the target film are attributed to the presence of the GeO_x_ interlayer, which promotes structural ordering and grain alignment. Grain size analysis based on FE‐SEM images indicates that the average grain size increased from 1.2 µm for the control film to 3.3 µm for the target film (Figure S11, Supporting Information). To complement the FE‐SEM observations and further investigate surface morphology, atomic force microscopy (AFM) was conducted on both the control and target films (Figure S12, Supporting Information). According to the AFM measurements, the control film exhibits greater surface roughness and a higher grain boundary density than the target film. This smoother topography with less pinholes of the target film underscores the role of the GeO_x_ layer in promoting film uniformity of SnS absorber which potentially forms a better interface with CdS buffer layer.

To investigate the effect of the substrate on the crystal structure and growth orientation of the VTD‐grown SnS films, XRD measurements were performed on samples deposited on the control and target substrates (Figure 3c). Both films reflect similar peaks that correspond well with the orthorhombic SnS (JCPDS card No. 00‐39‐0354) without any detectable secondary phase. In particular, the target film presents a pronounced increase in the intensity of the (101) and (111) diffraction peaks compared to the control positioned at 2θ values of 30.5° and 31.5°, respectively. This enhancement suggests improved crystallinity and a preferential growth orientation along the (101) and (111) planes in the SnS film grown on the GeO_x_‐modified substrate. To quantitatively assess the preferred crystallographic orientation, the texture coefficient (TC) was calculated for both films using standard methodology, with reference to the JCPDS pattern (00‐39‐0354, Figure 3d). Notably, the target film exhibited TCs exceeding 1.5 along the (101) and (111) planes, indicating strong preferential orientation in these directions. A detailed explanation incorporating relevant references to substantiate our statement is added in Note S1b (Supporting Information). In SnS, weak van der Waals interactions between adjacent layers limit efficient charge transport along in‐plane orientations, particularly along the (040) crystallographic direction, due to restricted interlayer hopping.^[^ ^66^, ^67^ ^]^ In contrast, tilted or 3D stacking configurations, such as those along the (101) and (111) planes, enhance charge carrier mobility by promoting stronger orbital overlap and more efficient interlayer electronic coupling. The control film, however, exhibited a relatively random crystallographic orientation and lower TCs. These findings confirm that introducing a GeO_x_ interfacial layer not only enhances film uniformity but also promotes favorable crystallographic orientation for carrier transport. This synergistic improvement in structural properties is closely correlated with the superior PV performance of the target devices.

To examine the influence of the GeO_x_ interfacial layer on Na^+^ ion diffusion from the SLG substrate and to analyze the elemental distribution across the device stack, secondary‐ion mass spectroscopy (SIMS) depth profiling was performed on SnS/CdS films deposited on both the control and target substrates (Figure 3e,f). Here, SIMS measurements were conducted across the entire stack, from the CdS buffer layer to the underlying Mo contact, enabling a direct comparison of elemental profiles. In the target device, SIMS measurements detected Ge and O signals localized near the Mo layer, confirming the presence of the GeO_x_ interlayer. Minor Ge diffusion into the Mo layer was also observed, likely owing to the sputtering effect during SIMS profiling. The results revealed that the target device exhibited a substantially attenuated Na signal compared to the control, indicating that the GeO_x_ layer acts as an effective diffusion barrier, limiting excessive Na migration from the SLG substrate into the SnS absorber layer. This suppression of Na diffusion plays an important role in enhancing absorber quality and device performance. While controlled Na incorporation can promote grain growth and defect passivation, excessive Na doping can introduce trap states and degrade *J*_SC_ performance.^[^ ^68^ ^]^ In this study, the higher *J*_SC_ values of the target device can be attributed, at least in part, to the moderated Na content resulting from the GeO_x_ layer. Previous studies have shown that metal oxide passivation layers with thicknesses of 5–10 nm can effectively transport holes. In contrast, thinner Al_2_O_3_ layers (≈2 nm) are ineffective at transporting holes, although they successfully block Na diffusion. Thus, the GeO_x_ interfacial layer enables controlled Na^+^ diffusion, improving absorber quality while preventing the detrimental effects associated with Na over‐doping.^[^ ^43^, ^69^ ^]^

**Figure 4:**
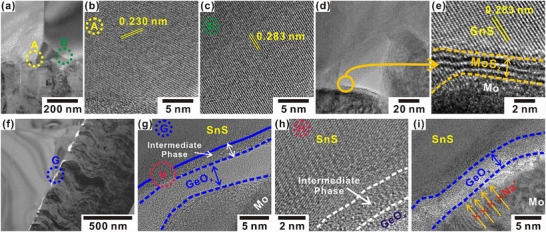
a) Low‐magnification cross‐sectional TEM image of the Mo/SnS interface in the control device. b,c) HR‐TEM images of regions A and B in (a). d) Mo/SnS interface. e) MoS_2_ at the Mo/SnS interface. f) Low‐magnification cross‐sectional TEM image of the Mo/GeO_x_ and GeO_x_/SnS interfaces in the target device. g) HR‐TEM image of region G in (f). h) Magnified HR‐TEM image of selected region H in (g). i) Mo/GeO_x_ interface and depiction of the blockage of Na diffusion from the SLG substrate into the SnS layer through the GeO_x_ layer.

To better understand the grain orientation and interfacial characteristics at the rear interface, transmission electron microscopy (TEM) analysis was performed on both control and target devices. The cross‐sectional TEM image of the control device (**Figure** **4**a) presents a compact and well‐adhered SnS layer on Mo back contact. Figure S13a,b (Supporting Information) shows the cross‐sectional TEM image of control and target devices up to SnS layer. High resolution TEM (HR‐TEM) images of selected regions A and B (Figure 4b,c), near the Mo/SnS interface, exhibit well‐defined SnS lattice fringes with spacings of 0.230 nm for the (101) plane and 0.283 nm for the (111) plane. No discernible secondary phases are observed at the grain boundaries, indicating high crystal quality. Further inspection of the Mo/SnS interface in the control device (Figure 4d) reveals ≈3‐nm‐thick MoS_2_ interfacial layer (Figure 4e), likely formed during the high‐temperature VTD process. Notably, such MoS_2_ formation can introduce additional series resistance and hinder charge extraction, thereby degrading device performance. In contrast, the target device modified with a GeO_x_ interfacial layer exhibits a distinct interfacial structure. Its cross‐sectional image (Figure 4f) presents layered stacking of constituent films at the back interface. The HR‐TEM image of region G (Figure 4g) highlights a conformal GeO_x_ interlayer, ≈5–7 nm thick, positioned between the Mo and SnS layers, along with ≈2 nm thick intermediate phase. Energy dispersive X‐ray spectroscopy (EDS) elemental mapping of the Mo/GeO_x_/SnS region (Figure S14, Supporting Information) affirms the spatial distribution of Mo, Ge, Sn, and O elements, with minimal Ge diffusion extending a few nanometers into the adjacent layers. The EDS mapping also confirms the presence of an intermediate phase within the GeO_x_ interlayer. This intermediate region may be a mixed phase of SnO_x_ and GeO_x_ (Figure 4h; Figure S14, Supporting Information), likely formed through interdiffusion and the reaction of Sn with oxygen within the GeO_x_ matrix during high‐temperature SnS growth, as reported in previous studies.^[^ ^70^, ^71^ ^]^ TEM analysis further confirmed that the GeO_x_ thickness is in approximation of calculated value in Note S1a (Supporting Information). At the GeO_x_/SnS interface, the presence of a mixed GeO_x_ and SnO_x_ phase was identified, with elemental mapping revealing its extension over ≈2 nm region. Such a controlled, graded interlayer with limited interfacial mixing provides a chemically stable, thermally robust, and electronically benign back‐contact passivation scheme. Another critical observation related to the target device is the absence of the MoS_2_ layer at the Mo/SnS interface, which is attributed to the introduction of the GeO_x_ interfacial layer. The formation of MoS_2_ between the SnS and Mo layers creates a Schottky barrier, restricting hole transport across the SnS/Mo interface.^[^ ^72^, ^73^, ^74^ ^]^ Thus, the suppression of MoS_2_ formation reduces interfacial resistance and improves charge collection. Furthermore, HR‐TEM images (Figure 4i) reveal localized elemental redistribution near the GeO_x_ interface, particularly within the Mo layer. To validate this observation, elemental mapping analysis was performed on a selected region (Figure S15a,b, Supporting Information), distinctly indicating the main presence of Na below the GeO_x_ layer. This spatial confinement of Na suggests that the GeO_x_ layer effectively acts as a diffusion barrier, inhibiting the upward migration of Na⁺ ions. These findings are consistent with the SIMS profiling, confirming the role of the GeO_x_ interfacial layer in effectively blocking excessive Na^+^.

**Figure 5:**
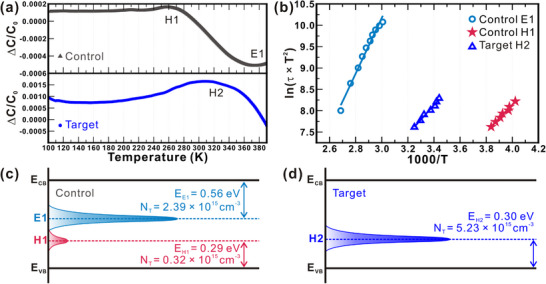
DLTS characterization of SnS devices with and without a GeO_x_ diffusion barrier. a) Normalized DLTS spectra showing defect levels E1 and H1 in the control device, and only H2 in the target device. b) Arrhenius plots for extracted trap activation energies. c,d) Schematic band diagrams indicating the position of trap levels with respect to the conduction and valence bands.

To investigate the impact of Na diffusion and the role of a GeO_x_ interfacial layer in modifying defect states in SnS TFSCs, deep‐level transient spectroscopy (DLTS) was carried out on two device architectures: the control device without GeO_x_ and the target device incorporating a GeO_x_ interlayer at the rear interface. **Figure** **5**a presents the DLTS spectra collected over a temperature range of 100–390 K. In the control device, the DLTS signal reveals two well‐resolved peaks. The first, denoted as E1 (minority carrier (electron) defect), appears at ≈377 K, while the second, H1 (majority carrier (hole) defect), is centered near 295 K. In contrast, the target sample exhibits only a single prominent peak, labeled H2, occurring at 307 K. To determine the thermal activation energy and capture cross‐sections associated with each observed trap, Arrhenius analysis was performed using the temperature‐dependent emission rates, as shown in Figure 5b. Linear fitting yielded activation energies of 0.56 and 0.29 eV for the E1 and H1 traps, respectively, in the control device, and 0.30 eV for the H2 trap in the target device. These values are indicative of both deep and relatively shallow defect levels within the SnS bandgap. The energetic position of each trap level with respect to the conduction band minimum (CBM) and valence band maximum (VBM) is summarized in the energy level diagrams shown in Figure 5c,d. The control device features both a deep‐level defect (E1) near mid‐gap and a shallower level (H1) close to the VBM. Meanwhile, the target device only exhibits the shallow trap (H2), suggesting that the deeper E1 level is suppressed upon incorporation of the GeO_x_ interlayer. These diagrams visualize the defect energy landscape and provide insight into how Na ion incorporation, or its inhibition via GeO_x_, alters the dominant recombination pathways in the absorber layer.

Quantitative analysis of the trap properties is summarized in **Table** **2**. For the control device, the trap density of E1 is 2.39 × 10^15^ cm^−3^, while H1 has a density of 0.32 × 10^15^ cm^−3^. Their respective capture cross‐sections are 4.43 × 10^−23^ cm^2^ and 6.62 × 10^−24^ cm^2^. In the target device, the H2 trap shows a higher density of 5.23 × 10^15^ cm^−3^ and a capture cross section of 5.15 × 10^−24^ cm^2^, which is comparable to that of the H1 trap in the control device. These values reflect a significant suppression of deep‐level traps and a redistribution of shallow defects in response to the GeO_x_ interfacial modification. From these observations, the shallow‐level defects (H1 and H2), which appear at nearly identical energies and capture cross‐sections, are attributed to Sn vacancies (V_Sn_), intrinsic to the SnS absorber.^[^ ^75^ ^]^ The remaining E1 defect, present exclusively in the control device, is attributed to Na_Sn_ anti‐site defects introduced via Na interstitials (Na_i_) diffusion from the SLG substrate and incorporation with V_Sn_.^[^ ^76^ ^]^ The absence of this signal in the target sample confirms that the rear interface passivation layer effectively prevents Na ion interdiffusion into the SnS bulk, thereby eliminating the formation of deep donor‐like traps. The elevated density of H2 in the target sample is interpreted as a consequence of the absence of Na_i_ passivation, resulting in more electrically active V_Sn_ sites. Notably, the deep‐level E1 defect in the control sample, located near mid‐gap, acts as a strong non‐radiative recombination center. Its presence is likely a key factor contributing to the reduced PV performance observed in the control device, as it accelerates carrier recombination and shortens carrier lifetimes. In contrast, the target device benefits from the elimination of this deep trap, pointing to the effectiveness of back interface engineering in improving optoelectronic quality by modulating defect chemistry.

**Table 2:** Summary of activation energies, trap densities, capture cross sections, and defect assignments.

	Activation Energy [eV]	Trap density [cm^−3^]	Capture cross‐section [cm^2^]	Defect identification
Control	0.56	2.39 × 10^15^	4.43 × 10^‒23^	Na_Sn_
	0.29	0.32 × 10^15^	6.62 × 10^‒24^	V_Sn_
Target	0.30	5.23 × 10^15^	5.15 × 10^‒24^	V_Sn_

**Figure 6:**
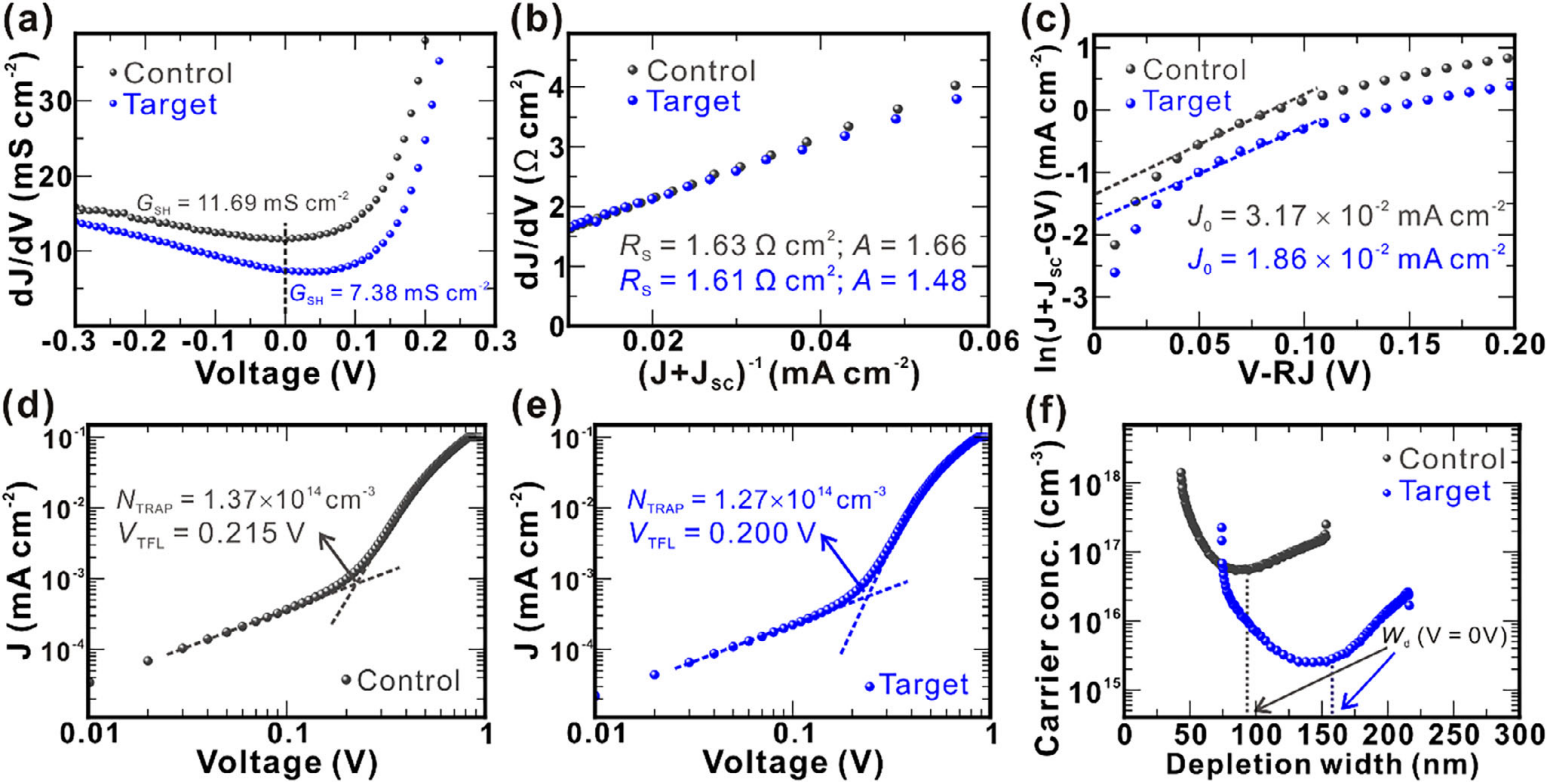
a) *G*_SH_, b) *R*_S_, and c) *J*_0_ plots for the control and target devices, extracted from dark *J*–*V* measurements. d,e) Full logarithmic plots of dark *J*–*V* curves for the control and target devices, respectively, with indicated *V*_TFL_ and *N*_TRAP_ and f) free carrier concentration as a function of depletion width, calculated from *C*–*V* measurements, for the control and target devices.

To assess the PV performance of the SnS absorbers deposited on the control and target substrates, dark *J*–*V* measurements were performed on the respective best‐performing devices (Figure S16a, Supporting Information). The extracted diode parameters offer important insights into the performance enhancement observed for the target device. Notably, the target device exhibits lower shunt conductance (*G*_SH_) than the control device (**Figure** **6**a), indicating improved shunt resistance and overall device quality. This improvement is attributed to enhancements in the morphological and crystalline properties of the absorber layer, facilitated by the GeO_x_ interfacial layer. Moreover, a reduced ideality factor (*A*) and lower series resistance (*R*_S_) in the target device (Figure 6b) suggest suppression of trap‐assisted recombination within the bulk and at the interface. The dark saturation current density (*J*_0_) is also lower in the target device compared to the control (Figure 6c), confirming a reduction in non‐radiative recombination losses associated with deep‐level trap states in the absorber. The diode parameters of both the target and control devices are summarized in Table S3 (Supporting Information). To gain deeper insights into trap dynamics, trap‐filled limit voltage (*V*_TFL_) and trap density (*N*_TRAP_) were determined through a space‐charge‐limited current analysis. Notably, the *V*_TFL_ and *N*_TRAP_ values of the target device were marginally lower than those of the control (Figure 6d,e; Note S2, Supporting Information). The lower trap density in the target device reaffirms that incorporating the GeO_x_ interlayer reduces defect states within the SnS absorber, thereby mitigating carrier trapping and recombination. Next, capacitance‐voltage (*C‒V*) profiling (Figure S16b and Note S3, Supporting Information) was performed to investigate interfacial charge dynamics. Figure 6f presents the carrier concentration distribution with respect to depletion width (*W*_d_). The lower carrier concentration in the target device (5.26 × 10^15^ cm^‒3^) compared to the control device (2.39 × 10^17^ cm^‒3^) could be attributed to GeO_x_‐induced suppression in excessive Na^+^ diffusion. Moreover, the *W*_d_ of the target device (158 nm) was substantially greater than that of the control (97 nm), attributed to the GeO_x_‐induced suppression of Na^+^ diffusion from the SLG substrate into the SnS absorber. While moderate Na^+^ diffusion can increase carrier concentration, excessive incorporation introduces deep‐level defect states that trap photogenerated carriers near the back interface, leading to device performance degradation.^[^ ^68^, ^76^ ^]^ In the target device, the GeO_x_ interlayer acts as an effective diffusion barrier, mitigating these effects and improving both the bulk and interfacial quality of the SnS absorber. The target device demonstrates a PCE of 4.81% compared to 3.71% for the control. However, the *V*_OC_ remains limited to 0.319 V, underscoring a persistent deficit and highlighting the need for targeted interface engineering and defect passivation strategies. To enhance *V*_OC_, recombination should be suppressed by optimizing heterojunction band alignment (via tailored buffer layers such as Zn(O, S), In_2_S_3_, or Zn_x_Sn_1‐x_O), while bulk and interfacial defects are mitigated through S‐rich growth, alkali/Cl‐assisted passivation, and rear‐interface engineering. *FF* improvements rely on contact stack engineering (barrier‐less or diffusion‐buffered back contacts), absorber texture optimization, and balancing thickness for transport versus absorption. Enhancing *J*_SC_ involves mild S/Se alloying for bandgap tuning, and minimizing parasitic absorption through thinner, higher‐quality window layers. We believe the interfacial approach initiated here provides a practical platform to implement for selective contacts and buffer alignment, which directly addresses the *V*_OC_ deficit. To evaluate device stability, we assessed its performance three months after fabrication without any encapsulation. As depicted in Figure S17 (Supporting Information), the device retained more than 96% of its initial efficiency, demonstrating excellent long‐term stability. Detailed PV parameters at the time of fabrication and after three months are summarized in Table S4 (Supporting Information).

## 3 Conclusion

In this study, we present a straightforward approach to rear‐interface passivation in VTD‐grown SnS TFSCs by forming a GeO_x_ layer through controlled oxidation of thermally evaporated Ge on SLG/Mo substrates. This GeO_x_ interlayer improves the uniformity and crystallinity of the SnS absorber while simultaneously suppressing deep‐level defects, blocking excessive Na⁺ diffusion from the SLG, and inhibiting the formation of a detrimental MoS_2_ interfacial layer. DLTS analysis provides direct evidence of this effect, revealing the elimination of a Na‐induced deep electron trap (E1) and a corresponding reduction in non‐radiative recombination. Together, these improvements significantly reduce recombination losses, enabling efficient carrier collection and delivering a PCE of 4.81%, one of the highest values reported for SnS‐based TFSCs. Nevertheless, the large *V*_OC_ deficit highlights the continuing need for interface and defect passivation strategies, including buffer‐layer band alignment such as Zn(O, S), In_2_S_3_, or ZTO, alkali/Cl‐assisted bulk passivation, and rear‐interface engineering. Further optimization of contact stacks and absorber texture could improve *FF*, while mild S/Se alloying and high‐quality, thinner window layers offer promising routes to enhance *J*_SC_. We believe the interfacial approach initiated here provides a practical platform to implement for selective contacts and buffer alignment, which directly addresses the *V*_OC_ deficit.

## 4 Experimental Section

### Ge Thin Film Deposition and Device Fabrication

A 7‐nm‐thick Ge thin film was deposited onto SLG substrates pre‐coated with an 800 nm thick Mo layer using thermal evaporation. Prior to Ge deposition, the SLG/Mo substrates were cleaned sequentially in isopropyl alcohol (IPA) and deionized water, each for 20 min in an ultrasonic bath. The cleaned substrates were then dried under a nitrogen gas flow and vacuum‐sealed until further use. Each substrate measured 2.5 cm × 2.5 cm. The base pressure of the evaporation chamber was maintained at 5 × 10^−6^ Torr, while the deposition rate was set at ≈0.2 Å s^‒1^ by adjusting the supplied current. Ge thin films with thicknesses ranging from 1–10 nm were also deposited onto SLG/Mo substrates.

### Controlled Oxidation of the Ge Thin Films

Although Ge naturally forms a native oxide upon exposure to air, controlling its oxidation process was deemed essential to achieve a uniform and stable GeO_x_ layer. To control native oxide formation, the deposited Ge thin films were subjected to high‐vacuum heat treatment under a steady flow of high‐purity oxygen (20 sccm). The base pressure of the chamber was maintained at 5 × 10^−6^ Torr. Meanwhile, the temperature was ramped to 240 °C at a rate of 10 °C min^‒1^, held for 2 min, and then allowed to fall naturally to room temperature (Figure 1a). The oxidation process was optimized in two stages: initially, the temperature was varied from 200–290 °C while maintaining a fixed 2 min duration; subsequently, the optimized temperature was fixed at 260 °C, and the duration was varied from 1–10 min. Following oxidation, the GeO_x_‐modified substrates were used for characterization and device fabrication.

### SnS Deposition and Device Fabrication

The SLG/Mo/GeO_x_ films subjected to controlled oxidation were used as substrates for the deposition of SnS thin films via VTD. The VTD chamber was first purged with high‐purity N_2_, and the substrates were placed on a flat quartz holder located at the upstream end of the heating zone. The system pressure was initially reduced to 5 mTorr and then stabilized at 2 Torr with Ar supply, which was maintained throughout the deposition. The furnace temperature was ramped to 600 °C at 20 °C min^‒1^, held for 20 min under constant pressure to facilitate SnS film growth, and then allowed to drop naturally to room temperature. The given deposition rate to ensure the cube‐like morphology and minimize the plate‐like growth of inherent orthorhombic SnS was used.^[^ ^9^, ^10^ ^]^ The temperature ramping rate during VTD growth was found to play a decisive role in governing the microstructure and optoelectronic quality of SnS films. At a slow ramping rate of 5 °C min^‒1^, the absorbers exhibited a nonuniform crystallite size distribution leading to inferior morphology and limited device performance. Increasing the ramping rate to 20 °C min^‒1^ promoted the formation of densely packed, cube‐like grains with a more favorable (120) orientation and improved film uniformity. These structural improvements reduced shunt pathways and enhanced charge transport, as reflected in the higher *FF* and overall device efficiency. Thus, in addition to conventional growth parameters such as pressure and temperature, the temperature ramping rate emerges as a key factor in tailoring SnS film quality and optimizing solar cell performance. The resulting films were then used to fabricate TFSCs and subjected to comprehensive characterization. To ensure deposition fidelity and prevent cross‐contamination, the furnace tube was cleaned using IPA after each run and baked at 700 °C for 60 min under a 160 sccm Ar flow to remove residual material from the chamber walls. After SnS absorber deposition, a CdS n‐type buffer layer was grown using the conventional chemical bath deposition method to form the heterojunction. The precursor solution was prepared and mixed at 8 °C, followed by CdS deposition in a recirculating bath at 60 °C for 22 min. After this buffer layer deposition, ≈50 nm thick i‐ZnO layer and ≈350 nm thick AZO layer were sequentially deposited via radiofrequency sputtering. The top metal contact, composed of aluminum, was deposited using direct current sputtering. The completed devices were divided into six individual cells using a patterned shadow mask, with each cell featuring an active area of 0.30 cm^2^.

### Film and Device Characterization

The crystallographic properties of the GeO_x_ and SnS films were characterized using a GI‐XRD system equipped with a PANalytical X'Pert PRO diffractometer operating at a wavelength of 1.5418 Å (Cu‐K_α_). The oxidation state of the GeO_x_ thin film was confirmed through XPS using an Mg‐K_α_ radiation source (1253.6 eV). The surface and cross‐sectional morphologies of the SnS absorber were examined using an FE‐SEM system (ZEISS GeminiSEM 500). The topography of the films was obtained using AFM analysis (PSIA, XE‐100). Elemental depth profiles were acquired through SIMS using a CAMECA IMS 7f magnetic sector instrument, employing 6 kV primary Cs⁺ ions over a scanned area of 200 × 200 µm^2^. To validate the presence of GeO_x_, TEM imaging was performed using a JEM‐ARM300F2 (JEOL, Japan) microscope. Sample preparation for this TEM analysis was conducted using a focused ion beam system (NX5000 Hitachi). The PV performance of the fabricated devices was examined using a Class AAA solar simulator (San‐ei Electric, XES‐301S), equipped with a Keithley 2400 source meter, under 1‐sun illumination (100 mW cm^−2^). Heterojunction properties were evaluated through dark *J–V* measurements using a semiconductor parameter analyzer (HP 4155B). EQE spectra were recorded at room temperature across the wavelength range of 300–1300 nm using a spectral response system (Jasco, CEP‐25BX) at the Energy Convergence Core Facility, Chonnam National University.

### DLTS Measurement

DLTS characterization was conducted using a cryogenic probe station (Lakeshore TTPX) integrated with a precision impedance analyzer (Zurich Instruments MFIA). Measurements were performed over a temperature range of 100 to 390 K, with a step increment of 1 K. At each temperature point, ten consecutive readings were averaged to enhance signal accuracy. *C–V* measurements were acquired at a fixed frequency of 60 kHz to evaluate the doping profile and depletion region characteristics of the device. During DLTS acquisition, a voltage pulse sequence was applied, sweeping from −1.0 to 0 V with a pulse duration of 100 ms and a repetition interval of 900 ms. The technique monitors the transient response of junction capacitance following the voltage pulse, capturing the thermal emission of carriers from trap states. This transient behavior enables the extraction of key defect parameters, including activation energy, capture cross‐section, and defect density.

## Conflict of Interest

The authors declare no conflict of interest.

## Author Contributions

R.K.Y. conceptualized the study, designed the experimental plan, fabricated the solar cells, performed device characterization, and drafted the manuscript. V.M. drafted the manuscript and contributed to data analysis. Y.T.K. conducted formal analysis and assisted with device characterization. G.U.K. deposited the Ge thin films. W.J. conducted DLTS measurement and data analysis. P.R.P. and N.B. assisted with formal analysis and data interpretation. J.H.K. provided critical insight during manuscript drafting. Y.Y. provided critical insight during DLTS measurement and data interpretation. J.H. received the funds, supervised the project, and co‐drafted the manuscript.

## Supporting information



Supporting Information

Supporting Information

## Data Availability

The data that support the findings of this study are available from the corresponding author upon reasonable request.
