# Supplementary material for: Optimized Rear‐Interface Passivation of SnS Thin‐Film Solar Cells Using a Controlled Germanium Oxide Interlayer for Enhanced Photovoltaic Performance
Source: Small. 2025 Sep 19;21(51):e07626. doi: 10.1002/smll.202507626 (PMC12723332; doi:10.1002/smll.202507626)
Supplement: Supplementary file 1 — Supporting Information [file SMLL-21-e07626-s002.docx]

Supporting Information

Optimized Rear-Interface Passivation of SnS Thin-Film Solar Cells Using a Controlled Germanium Oxide Interlayer for Enhanced Photovoltaic Performance

Rahul K. Yadav^1^, Vishesh Manjunath^1^, Yong Tae Kim^1^, Girish U. Kamble^1^, Wookyung Jeon^2^, Parag R. Patil^1^, Neha Bisht^1^, Jin Hyeok Kim^1^, Yohan Yoon^2^, and Jaeyeong Heo^1,^*

^1^Department of Materials Science and Engineering, and Optoelectronics Convergence Research Center, Chonnam National University, Gwangju 61186, Republic of Korea

^2^ Department of Materials Engineering, Korea Aerospace University, Goyang 10540, Republic of Korea

***Corresponding Author:**

E-mail address: jheo@jnu.ac.kr

**Supplementary Note 1a**

Using the mass–density relation,

$t = \frac{m}{\rho A}$ (1)

and accounting for the molar volume change upon oxidation

Ge (ρ = 5.32 g cm⁻³, M = 72.63 g mol^‒1^)

GeO_x_ (~ρ = 4.1 g cm^‒3^, M = 104.5 g mol^‒1^),

The calculated volume expansion factor is ~1.36. Thus, oxidation of a 7 nm Ge layer would theoretically yield ~9.5 nm of GeOₓ, consistent with the experimentally observed thickness increase of ~2.5 nm.

**Supplementary Note 1b**

To calculate the specific orientation of crystal planes in the control and target film fabricated using substrate with different rear interface properties, the texture coefficient (TC) was calculated with the measured XRD and standard JCPDS using below equation:

$TC(hkl)= \frac{\frac{I(hkl)}{I_{0}(hkl)}}{\left( \frac{1}{N}\sum_{i=1}^{N} \frac{I(h_{i}k_{i}l_{i})}{I_{0}(h_{1}k_{1}l_{1})} \right)}$ (1)

where *I(hkl)* is the diffraction peak intensity corresponding to the measured XRD pattern, *I*_0_*(hkl)* is the peak intensity in the standard (JCPDS) pattern, and *N* is the total number of planes considered in the measurement.

The degree of preferred orientation was further evaluated from the standard deviation σ of all TC values, calculated as:

*σ =* $\sqrt{\sum\frac{1}{n}{(TC(hkl)-1)}^{2}}$ (2)

A lower σ value indicates a more random orientation, whereas higher values reflect stronger preferential orientation. For a particular plane, TC values in the range of approximately 1.45–1.50 are generally considered indicative of a preferred orientation, as supported by literature.^[1]^

**Supplementary Note 2**

The full logarithmic *J‒V* characteristics of the control and target devices are used to estimate the trap characteristics of the devices, the space-charge-limited current (SCLC) model was applied. Trap field voltage (*V*_TFL_) was estimated using full logarithmic *J–V* characteristics. The corresponding trap density (*N*_TRAP_) was estimated using the following equation:

$N_{TRAP}= \frac{2\varepsilon_{r}\varepsilon_{0}V_{\mathrm{TFL}}}{eL^{2}}$ (2)

where 𝜀_r_ is the relative permittivity of SnS (12.5) ^[2, 3]^, 𝜀_0_ is the permittivity of vacuum, *e* is the electronic charge, and *L* is the thickness of the SnS thin film (1.5 µm).

**Supplementary Note 3**

The carrier concentration, built-in potential (*V*_bi_), and depletion width of control and target devices are measured by the *C–V* profiling. The extrapolation of plots of C^‒2^ *vs*. V gives *V*_bi_. The carrier concentration and depletion width were obtained by the following equations

$N_{a}=\frac{-2}{q\varepsilon_{r}\varepsilon_{0}A^{2}\frac{dC^{-2}}{dV}}$ (3)

$W_{d}=\frac{q\varepsilon\varepsilon_{0}A}{C_{0}}$ (4)

where 𝜀_r_ is the relative permittivity of SnS (12.5) ^[2-5]^, 𝜀_0_ is the permittivity of vacuum, and *A* is the active area (0.3 cm^2^) of the cell. *C*_0_ is the fitting parameter derived from *C–V* curve.

**Supplementary Figures**

**Figure S1** Average photovoltaic (PV) parameters of the thin-film solar cells (TFSCs) fabricated using the SLG/Mo/Ge back contact configuration at different Ge thickness: (a) open circuit voltage (*V*_OC_), (b) short circuit density (*J*_SC_), (c) fill factor (*FF*), and (d) power conversion efficiency (PCE).

**Figure S2** Light *J‒V* characteristics of the thin-film solar cells (TFSCs) fabricated using the SLG/Mo/Ge back contact configuration at a Ge thickness of (a) 1 nm, (b) 3 nm, (c) 5 nm, (d) 7 nm, and (e) 10 nm.

**Figure S3** Light *J‒V* curve of the best cell from devices fabricated with and without Ge interfacial layer at the back contact (Mo/SnS, and Mo/Ge/SnS configurations).

**Figure S4** Top surface, cross-section, and schematic of (a) Mo substrate, and (b) Mo/Ge substrate

**Figure S5** (a) GI**-**XRD pattern of SLG/Mo, SLG/Mo/Ge (uncontrolled oxidation) substrate. (b) zoomed to check for any possible peaks of Ge.

**Figure S6** (a) XPS 3d peaks of Ge at the top surface with O 1s peak of Mo/Ge (uncontrolled oxidation) substrate. (b) XPS 3d peaks of Ge after 5 nm etching with O 1s peak of Mo/Ge (uncontrolled oxidation) substrate.

**Figure S7** PV performance with temperature optimization of controlled oxidation of Mo/GeO_x_ (controlled oxidation) of the target device. (a) *V*_OC_, (b) *J*_SC_, (c) *FF*, and (d) PCE.

**Table S1** PV parameters with temperature optimization during controlled oxidation for a duration of 2 min.

| **Oxidation Temp.** | **PV parameters** | | | |
| --- | --- | --- | --- | --- |
|  | *V*_OC_ (V) | *J*_SC_ (mA cm^-2^) | *FF* (%) | PCE (%) |
| 200 °C | 0.290 | 20.36 | 41.9 | 2.48 |
| 230 °C | 0.327 | 25.31 | 50.1 | 4.16 |
| 260 °C | 0.337 | 22.28 | 56.4 | 4.24 |
| 290 °C | 0.289 | 22.33 | 32.5 | 2.10 |

**Figure S8** PV performance with time optimization of controlled oxidation of Mo/GeO_x_ (controlled oxidation) of the target device. (a) *V*_OC_, (b) *J*_SC_, (c) *FF*, and (d) PCE.

**Table S2** PV parameters with time optimization during controlled oxidation at a fixed temperature of 260 °C.

| **Oxidation time** | **PV parameters** | | | |
| --- | --- | --- | --- | --- |
|  | *V*_OC_ (V) | *J*_SC_ (mA cm^-2^) | *FF* (%) | PCE (%) |
| 1 min | 0.342 | 23.04 | 51.1 | 4.03 |
| 3 min | 0.337 | 24.32 | 52.8 | 4.34 |
| 5 min | 0.319 | 27.00 | 56.0 | 4.81 |
| 7 min | 0.333 | 24.79 | 55.6 | 4.56 |
| 10 min | 0.309 | 20.40 | 49.3 | 3.11 |

**Figure S9** Light *J‒V* characteristics of the best cells of devices with (a) temperature optimization and (b) time optimization of controlled oxidation of Mo/GeO_x_ (controlled oxidation) target substrate.

**Figure S10** (a) XPS O 1s peak at the top surface of Mo/GeO_x_ (controlled oxidation) target substrate.

**Figure S11** The fitted grain size distribution is based on the Gaussian function (a) Mo/SnS control device, (b) Mo/GeO_x_ (controlled oxidation)/SnS target device.

**Figure S12** AFM topography (area of 10 × 10 μm^2^) of the SnS thin films at (a) Mo/SnS control device, (b) Mo/GeO_x_ (controlled oxidation)/SnS target device.

**Figure S13** Cross-sectional TEM image of (a) control, and (b) target devices**.**

**Figure S14** Elements mapping analysis of the selected area from cross-sectional TEM (a) Sn, (b) S, (c) Mo, (d) O, (e) Ge, and (f) cross-sectional TEM image of focused area.

**Figure S15** (a) Magnified HR-TEM image of selected area from Figure 4i, (b) Elemental mapping (traces of Na) of the area under the GeO_x_ layer.

**Figure S16** (a) Semi logarithmic plot of dark *J‒V* characteristics, (b) *C‒V* profiling of control and target devices**.**

**Table S3** Diode parameters of best performing devices with (target) and without (control) back contact modification, estimated from dark the *J‒V*.

| **Device** | ***R*_S_**  (Ω-cm^2^) | ***G*_SH_**  (mS cm^-2^) | ***J*_0_**  (× 10^-2^ mA cm^-2^) | ***A*** |
| --- | --- | --- | --- | --- |
| Control | 1.63 | 11.69 | 3.17 | 1.66 |
| Target | 1.61 | 7.38 | 1.86 | 1.48 |

**Figure S17** Stability test of the device after 3 months of fabrication.

**Table S4** PV parameters of the champion cell of target devices with modified back interfaces at the initial stage and after 3 months of fabrication.

| **Duration** | **Cell Performance** | | | |
| --- | --- | --- | --- | --- |
|  | *V*_OC_ (V) | *J*_SC_ (mA cm^-2^) | *FF* (%) | PCE (%) |
| Initial | 0.319 | 27.00 | 56.0 | 4.81 |
| After 3 months | 0.320 | 26.76 | 54.1 | 4.64 |

References

[1] G. Zoppi, N. S. Beattie, J. D. Major, R. W. Miles, I. Forbes, *J. Mater. Sci.* **2011**, 46, 4913.

[2] B. D. Malone, A. Gali, E. Kaxiras, *Phys. Chem. Chem. Phys*. **2014**, 16, 26176.

[3] H. T. Nguyen, V. L. Le, T. M. H. Nguyen, T. J. Kim, X. A. Nguyen, B. Kim, K. Kim, W. Lee, S. Cho, Y. D. Kim, *Sci. Rep.* **2020**, 10, 18396.

[4] S. M. Sze, Y. Li, K. K. Ng, *Physics of semiconductor devices*, John wiley & sons, **2021**.

[5] T. Sajeesh, N. Poornima, C. S. Kartha, K. Vijayakumar, *Phys. Status Solidi A* **2010**, 207, 1934.
